# Implanted Progestin Causing Pain and Psychiatric Disturbances in Porphyria Attack: A Case Report

**DOI:** 10.5811/cpcem.1490

**Published:** 2023-07-03

**Authors:** Ryan K. Misek, Massimo F. Riitano

**Affiliations:** * Midwestern University, Chicago College of Osteopathic Medicine, Department of Emergency Medicine, Downers Grove, Illinois; † Midwestern University, Chicago College of Osteopathic Medicine, Downers Grove, Illinois

**Keywords:** case report, porphyria, hemin, urine porphyrins

## Abstract

**Introduction:**

Acute hepatic porphyrias (AHP) are a rare group of inherited disorders caused by abnormal functioning of the heme synthesis pathway. Patients often present with diffuse abdominal pain, neurologic dysfunction, and hyponatremia.

**Case Report:**

We present a case of a 25-year-old female who presented with AHP after implantation of progestin birth control. The patient was confused, markedly tachycardic and hypertensive, and complained of severe abdominal pain. Spot urine ordered during the emergency department workup was later found positive for porphyrins and porphobilinogen (PBG).

**Conclusion:**

Acute hepatic porphyrias typically present with nonspecific symptoms in young women and are often overlooked in the acute care setting. Spot urine testing for PBG and urine porphyrins should be initiated early in patients with clinical suspicion of AHP.

## INTRODUCTION

Acute hepatic porphyrias (AHP) comprise a rare group of inherited disorders caused by abnormal functioning of the heme synthesis pathway.^1^ The dysfunction leads to the accumulation of metabolites and subsequent neurovisceral manifestations.^1^ Heme is essential in the function of the cytochrome P450 (CYP450) pathway. Induction via medications such as sulfonamides, anesthetics, anticonvulsants, or sex hormones can precipitate acute attacks.^1^ Patients often present with diffuse abdominal pain, neurologic dysfunction, and hyponatremia.^2^,^3^ We present a case of a 25-year-old female who presented with AHP after implantation of progestin birth control.

## CASE REPORT

A 25-year-old female with a history of recent severe acute respiratory syndrome coronavirus 2 infection presented to the emergency department (ED) with altered mental status and lower abdominal pain of several days’ duration. The patient was confused and agitated but was redirectable. The patient’s mother at bedside reported that her daughter had an episode of urinary incontinence and appeared to be delusional. She reported that the last time her daughter had acted similarly she had a supposed spider bite to her finger. The mother reported that the patient had fallen the previous day and struck her head. She also reported that the patient had a etonogestrel (a synthetic progesterone) birth control implant placed in her left arm 10 days prior. She endorsed inability to sleep. The patient denied any shortness of breath, constipation, back pain, fevers, or chills.

The physical examination was limited due to acute confusion, but vitals showed a blood pressure of 156/110 millimeters of mercury, a heart rate of 135 beats per minute, and respiration rate of 26 breaths per minute. The patient was noted to have a three-centimeter contusion to the right forehead. Abdominal examination revealed a soft and flat abdomen with intermittent voluntary guarding and no localizable tenderness, rebound, or rigidity.

Our review of the medical chart revealed that the patient had been seen at an outside hospital four days prior for similar complaints and discharged from the ED. Medical records showed she had a negative computed tomography (CT) of the abdomen and pelvis with intravenous (IV) contrast, negative transvaginal ultrasound with Doppler, unremarkable speculum exam, negative *Chlamydia trachomatis and Neisseria gonorrhoeae* deoxyribonucleic acid probe, normal vaginal wet mount, and unremarkable lab work. Her pain had been treated and she was discharged with dicyclomine, ondansetron, ibuprofen, and docusate sodium. She reportedly had difficulty urinating despite having the urge and drinking plenty of water.

The patient was given droperidol 1.25 milligrams (mg) IV and morphine 2 mg IV without relief. A set of CTs of her head, neck, and abdomen were negative for acute pathology. A qualitative urine drug screen was positive for cannabinoids at a screening cutoff of 50 nanograms per milliliter (mL). A clean catch urine sample was collected and found to be red in color. The specimen tube was wrapped in Coban to protect against light, and spot urine porphyrin was sent to an outside facility for analysis. Her sodium was 118 milliequivalents per liter (mEq/L) (reference range,135–145 mEq/L); 3% sodium chloride continuous IV infusion at a rate of 50 mL/hour (hr) was started after consultation with the nephrology service. She was admitted to the intensive care unit under the hospitalist service.

The patient remained acutely altered during her admission, and cardiology placed her on carvedilol 12.5 mg orally twice a day and clonidine 0.1mg/24hr transdermal patch to control her hypertension and tachycardia. She complained of weakness, and her creatine kinase was found to be over 5000 units/L (reference range, 30–145 U/L) on serial draws. Coronavirus disease 2019 antibody immunoglobulin (Ig) G was found to be positive, and the IgM was negative. An autoimmune panel was significant only for a slightly elevated anti-centromere antibody level. A work-up for pheochromocytoma via CT abdomen was negative. Magnetic resonance imaging with and without contrast did not reveal acute pathology. Cerebrospinal fluid was drawn via lumbar puncture, and testing for viral, bacterial, and fungal infections was negative.

**CPC-EM Capsule**

What do we already know about this clinical entity?

*Acute Hepatic Porphyria is a rare disorder caused by an inborn error of heme synthesis and presents with a unique constellation of non specific signs and symptoms.*

What makes this presentation of disease reportable?

*Acute Hepatic Porphyria has rarely been reported after implantation of progesterone birth control.*

What is the major learning point?

*How to recognize acute porphyria attacks and how to initiate proper work-up and treatment from the emergency department.*

How might this improve emergency medicine practice?

*Early treatment of acute porphyria spares the patient significant morbidity and diagnosis leads to prevention of recurrent attacks.*

The results of the spot urine porphyrin test, which were returned six days after admission, were significantly elevated as shown in Table 1. Concern was raised that the progesterone implant had triggered her attack, and it was removed the following day. The results of a 24-hr collection of urine porphyrin and porphobilinogen (PBG) (returned eight days after admission) were also elevated as shown in Table 2, which confirmed the diagnosis of AHP. The patient was started on dextrose 10% in water. Plasma porphyrins were also tested and demonstrate elevated plasma porphyrins of 34 nanomoles (nmol)/L (reference range, 0–15 nmol/L). The patient had returned to baseline mental status but was unable to ambulate. She was transferred to a tertiary care center where she received four hemin infusions at 4 mg per kilogram per day.

The patient had a prolonged hospital course after transfer for a total of 33 days inpatient. She had severe weakness and myopathy that prevented her from ambulating and required 14 days of inpatient physical therapy. She required 22 visits at outpatient physical therapy over the course of three months and subsequently suffered a second attack of porphyria five months after the initial presentation, which resolved with four days of hemin infusion. Genetic testing showed a positive heterozygous hydroxymethylbilane synthase pathologic mutation, which indicated a final diagnosis of acute intermittent porphyria (AIP).

## DISCUSSION

Acute hepatic porphyrias comprise a rare group of genetic diseases caused by altered heme biosynthesis. The four subtypes of AHP are AIP, variegate porphyria, hereditary coproporphyria, and delta-aminolevulinic acid dehydratase deficiency porphyria.^1^ The most common is AIP, with one carrier per 2,000 people in the Western population; it is inherited as an autosomal dominant mutation with 10% penetrance with an 80–90% female predominance.^4^ It typically presents in young women of reproductive age with abdominal pain, hyponatremia, fatigue, confusion, and stupor. Patients can experience hypertension and tachycardia that can be life-threatening.^1^–^3^ Insomnia is often an early symptom of an AIP attack.^5^ Metabolic stressors and cytochrome (CYP) P450 inducers, such as sulfonamides, alcohol, and sex hormones, can precipitate acute attacks.^6^ Variegate porphyria and HCP can also present with cutaneous blistering on light-exposed skin.^11^

Acute hepatic porphyria can present after implantation of progesterone birth control.^7^ Progesterone is thought to be responsible for cyclical attacks in women, but there is likely a multifactorial cause involving individual variation in progesterone metabolism and CYP450 activity. The risk for potential complications and benefits of hormonal therapy should be evaluated individually by a clinician, and hormone therapy should be stopped if patients begin to experience porphyria symptoms.^3^

Severe diffuse abdominal pain occurs in 90% of patients and in the presence of normal imaging and lab results, making diagnosis difficult.^8^ Since patients often have a history of repeat visits to the ED for undifferentiated abdominal pain where they receive opioid analgesics, they may be suspected of drug-seeking behavior. Cannabinoid hyperemesis syndrome is becoming increasingly prevalent and may obscure the clinical picture in patients who smoke marijuana.^9^ Accurate and timely diagnosis of porphyria prevents the development of long-term complications that include chronic kidney disease, chronic hypertension, hepatocellular carcinoma, polyneuropathy, depression, and anxiety.^8^,^10^

Current recommendations for diagnosis of patients suspected of AHP vary, but from the emergency physician’s standpoint, a spot urine collection for both PBG and total porphyrins is specific and sensitive enough to make the diagnosis.^11^,^12^ Twenty-four-hour collection of urine is no longer considered necessary for diagnosis and delays treatment. The sample container should be protected from light with a foil wrapping and frozen or refrigerated to protect against degradation of light-sensitive compounds. Urine porphyrin elevation occurs in many medical conditions and must be correlated clinically to rule in AHP. Urine discoloration is a common finding, but nonspecific. Second-line testing of plasma porphyrins with fluorescence or collection of fecal porphyrins can be conducted later to differentiate the AHP subtype.^11^

Symptom control of the acute attack consists of IV opioids, antiemetics, and anxiolytics. Beta-blockers are commonly used to prevent tachycardia, arrhythmia, and hypertensive crisis. Hemin therapy is the definitive treatment in moderate to severe attacks and should be started after the demonstration of typical symptoms of acute porphyria and elevation of urine PBG.^1^,^3^ Carbohydrate loading via dietary intake or IV infusions at 300–500 grams per day can be used during mild attacks or if hemin is not available locally, and patients receiving infusions should be closely monitored for hyponatremia. Any patient with an acute porphyria episode with worsening symptoms or lack of improvement within one to two days should receive hemin therapy.^1^,^3^,^8^ Prophylactic treatments for recurrent attacks include gonadotropin-releasing hormone agonists, IV hemin, and subcutaneous givosiran, which is a small interfering ribonucleic acid medication causing degradation of the delta-aminolevulinate synthase 1 enzyme.^11^ Our patient was advised to begin prophylactic hemin or givosiran therapy should she experience more than 4–6 attacks per year.

## CONCLUSION

We present a case of a patient experiencing worsening symptoms of AHP following progesterone birth control implantation. Acute hepatic porphyria is one of a rare group of diseases that typically present with nonspecific symptoms in young women and are often overlooked in the acute care setting.^2^ Spot urine testing for PBG and urine porphyrins should be initiated in patients with clinical suspicion of AHP.^11^,^12^ Symptoms are managed with IV analgesia, antiemetics, and beta-blockers. Timely treatment via correction of electrolyte imbalances and initiation of hemin therapy prevents significant morbidity and mortality.^1^,^3^,^8^

## Figures and Tables

**Table 1:** The patient’s lab test values collected on the day of admission show elevated porphyrins but were non-specific findings. When clinically correlated with her symptoms they increased the likelihood of an acute hepatic porphyria.

Spot Urine Porphyrins	Patient value	Reference range
Coproporphyrin-I urine-CRT ratio	39	0–6 μmol/mol
Coproporphyrin-III urine-CRT ratio	138	0–14 μmol/mol
Heptacarboxylate urine-CRT ratio	4	0–2 μmol/mol
Uroporphyrin urine-CRT ratio	435	0–4 μmol/mol

*CRT*, creatinine; *μmol/mol*, micromole per mole.

**Table 2:** The patient’s lab test values collected over a 24-hour period show elevated porphyrins. Significantly elevated porphobilinogen is highly sensitive and specific for acute hepatic porphyrias.

24-hr Urine porphyrins and porphobilinogen	Patient value	Reference range
Coproporphyrin-I urine-CRT ratio	21	0–6 μmol/mol
Coproporphyrin-III urine-CRT ratio	66	0–14 μmol/mol
Heptacarboxylate urine-CRT ratio	10	0–2 μmol/mol
Porphobilinogen 24 hr	617.4	0–11 μmol/mol
Porphobilinogen per volume	233.0	0–8.8 μmol/mol
Uroporphyrin urine-CRT ratio	173	0–4 μmol/mol

*CRT*, creatinine; *μmol/mol*, micromole per mole; *Hr*, hour.
